# The herpes simplex virus tegument protein pUL21 is required for viral genome retention within capsids

**DOI:** 10.1371/journal.ppat.1010969

**Published:** 2022-11-14

**Authors:** Ethan C. M. Thomas, Maike Bossert, Bruce W. Banfield

**Affiliations:** Department of Biomedical and Molecular Sciences, Queen’s University, Kingston, Ontario, Canada; University of Pittsburgh School of Medicine, UNITED STATES

## Abstract

During virion morphogenesis herpes simplex virus nucleocapsids transit from the nucleoplasm to the cytoplasm, through a process called nuclear egress, where the final stages of virion assembly occur. Coupled to nuclear egress is a poorly understood quality-control mechanism that preferentially selects genome-containing C-capsids, rather than A- and B-capsids that lack genomes, for transit to the cytoplasm. We and others have reported that cells infected with HSV strains deleted for the tegument protein pUL21 accumulate both empty A-capsids and C-capsids in the cytoplasm of infected cells. Quantitative microscopy experiments indicated that C-capsids were preferentially selected for envelopment at the inner nuclear membrane and that nuclear integrity remained intact in cells infected with pUL21 mutants, prompting alternative explanations for the accumulation of A-capsids in the cytoplasm. More A-capsids were also found in the nuclei of cells infected with pUL21 mutants compared to their wild type (WT) counterparts, suggesting pUL21 might be required for optimal genome packaging or genome retention within capsids. In support of this, more viral genomes were prematurely released into the cytoplasm during pUL21 mutant infection compared to WT infection and led to enhanced activation of cellular cytoplasmic DNA sensors. Mass spectrometry and western blot analysis of WT and pUL21 mutant capsids revealed an increased association of the known pUL21 binding protein, pUL16, with pUL21 mutant capsids, suggesting that premature and/or enhanced association of pUL16 with capsids might result in capsid destabilization. Further supporting this idea, deletion of pUL16 from a pUL21 mutant strain rescued genome retention within capsids. Taken together, these findings suggest that pUL21 regulates pUL16 addition to nuclear capsids and that premature, and/or, over-addition of pUL16 impairs HSV genome retention within capsids.

## Author summary

pUL21 is a conserved, multifunctional alphaherpesvirus protein involved in cell-to-cell spread of infection, transport of capsids along microtubules, and regulation of the phosphorylation status of both viral and host proteins. pUL21 thereby controls diverse processes such as nuclear egress of capsids and cellular lipid trafficking. This study provides additional insight into HSV-1 and HSV-2 pUL21 activities and suggests that, by binding pUL16, pUL21 prevents pUL16 from interacting prematurely with nuclear capsids that would otherwise lead to capsid destabilization and premature ejection of viral genomes. Thus, prevention of pUL21 interaction with pUL16 may prove to be a useful strategy for interfering with virion assembly.

## Introduction

The alphaherpesviruses herpes simplex virus (HSV) 1 and 2 are highly prevalent human pathogens [1,2]. Viral genome synthesis, capsid assembly, and genome packaging into capsids occur in the nuclei of infected cells. Capsid assembly begins with the formation of a spherical two-shelled structure called a procapsid [3]. The outer shell is comprised of 150 pentons and 11 hexons of the major capsid protein VP5, triplexes composed of proteins VP23 and VP19c that link the hexons and pentons, as well as one capsid portal composed of a dodecamer of pUL6, through which nascent genomes are packaged [3]. The inner shell is the capsid scaffold and is constructed from the large and small scaffolding proteins pUL26 and pUL26.5. Genome packaging is initiated by the association of the terminase complex with the portal triggering a signal transduction event that leads to the activation of pUL26 protease activity and subsequent release of the scaffold. This results in angularization of the capsid shell as the genome is packaged into the capsid. Successful genome packaging results in a genome-containing capsid referred to as a C-capsid. In addition to C-capsids, two by-products of capsid maturation, A- and B-capsids, are also produced in infected nuclei. B-capsids are thought to have initiated proteolytic cleavage of the scaffolding proteins and become angularized without engaging the terminase complex [4,5]. As such, B-capsids are terminal products that lack genomic DNA and contain a cleaved form of the small scaffolding protein, VP22a, trapped inside the capsid. A-capsids arise through unsuccessful genome packaging events or through the inability of the capsid to retain the viral genome [4–6]. Regardless of their origin, A-capsids are angularized and empty with no genomic DNA or scaffolding proteins present within their interior. In general, A-capsids are less abundant in infected cells compared to C-capsids as several mechanisms are utilized to retain the viral genome within capsids after packaging [3].

One of the most studied contributors to the retention of viral genomes within capsids are the capsid vertex specific component (CVSC) proteins pUL17, pUL25, and pUL36 [7–12]. These proteins are bound to triplexes adjacent to capsid vertices and are required for stabilizing the capsid structure against the high internal pressure exerted by the packaged genome [7,12–15]. While CVSC proteins are found on A-, B-, and C-capsids, they are more abundant on C-capsids [14,16]. pUL17 is important for genome packaging and the efficient recruitment of pUL25 and pUL36 to capsids [12,14]. pUL25 on capsid vertices prevents the capsid structure from rupturing after packaging [17,18]. Further, recent cryo-EM studies examining pUL25 and its β- and γ-herpesvirus homologs have demonstrated that pUL25 adjacent to the capsid portal is thought to extend over the portal, forming a pentameric portal cap that plugs the capsid portal after genome packaging [14,19–21]. pUL36 is important for the recruitment of the tegument to capsids during maturation and for proper portal plug conformation [9,14,22]. In addition to the CVSC proteins, capsids possess disulfide bonds that covalently link capsid proteins [23–25]. Impairing these complexes results in the dissociation of pentons, triplexes, and CVSC proteins from the capsid and the loss of genomes from the capsid. Ultimately, the successful packaging and retention of the genome into a newly assembled capsid is only one hurdle HSV capsids must face in the virion maturation process.

After genome packaging, C-capsids must transit from the nucleus to the cytoplasm for the final stages of virion assembly [26]. However, capsids are too large to pass through nuclear pore complexes. Thus, capsids undergo a specialized process conserved throughout the *Herpesviridae* family, called nuclear egress [26,27]. Nuclear egress is a complex and highly regulated process whereby C-capsids bud into the perinuclear space, acquiring a primary envelope derived from the inner nuclear membrane, followed by fusion of this envelope with the outer nuclear membrane, thereby releasing the capsid into the cytoplasm. Nuclear egress is also coupled to a poorly understood quality-control mechanism that preferentially selects C-capsids, rather than A- and B-capsids, to undergo primary envelopment at the inner nuclear membrane [28].

The capsid-associated tegument protein pUL21 is one of several viral proteins that function in nuclear egress and is conserved amongst the alphaherpesvirus subfamily [29–32]. pUL21 forms a tripartite complex in the mature HSV virion, interacting with tegument protein pUL16, which in turn interacts with pUL11 [33–37]. During the early stages of infection, pUL21 has been implicated in the trafficking of capsids to nuclei in HSV-1, HSV-2, and pseudorabies virus (PRV) infected cells as well as being required for optimal viral gene expression [31,38–40]. Late in infection, pUL21 regulates nuclear egress, tegument formation, cell-to-cell spread of infection and actives the ceramide transport protein, CERT [29,32,33,35,41–44]. Despite significant research into several pUL21 activities, the function pUL21 performs on capsids has yet to be thoroughly investigated. Previous studies from this laboratory and others have demonstrated that HSV-1 and HSV-2 pUL21 mutant strains accumulate both A- and C- capsids in the cytoplasm of infected cells [42,45]. In wild type (WT) virus infected cells, the majority of capsids found in the cytoplasm are C-capsids while A-capsids are almost exclusively found in the nucleus [3,16]. Currently, there is no explanation for the accumulation of A-capsids in the cytoplasm of cells infected with pUL21 deficient strains.

At least three hypotheses might explain the cytoplasmic accumulation of A-capsids in cells infected with pUL21 mutants. First, nuclear integrity may be disrupted in cells infected with pUL21 mutant strains leading to indiscriminate leakage of capsids from the nucleoplasm into the cytoplasm. Second, pUL21 may be required for the preferential selection of C-capsids for nuclear egress and its deletion may result in the selection of A-capsids for nuclear egress. Finally, it may be that viral genomes are not stably packaged into pUL21 mutant capsids resulting in premature expulsion of the genome from capsids once they reach the cytoplasm. The goal of this study was to evaluate these three hypotheses.

## Results

### Deletion of UL21 results in an increased abundance of nuclear and cytoplasmic A-capsids in infected cells

HSV-1 and HSV-2 pUL21 mutants (Δ21) accumulate both A- and C-capsids in the cytoplasm of infected cells [42,45]. Although this phenotype has been documented, quantification of A-capsids in HSV-1 and HSV-2 Δ21 infected cells has not been conducted.

**Fig 1:**
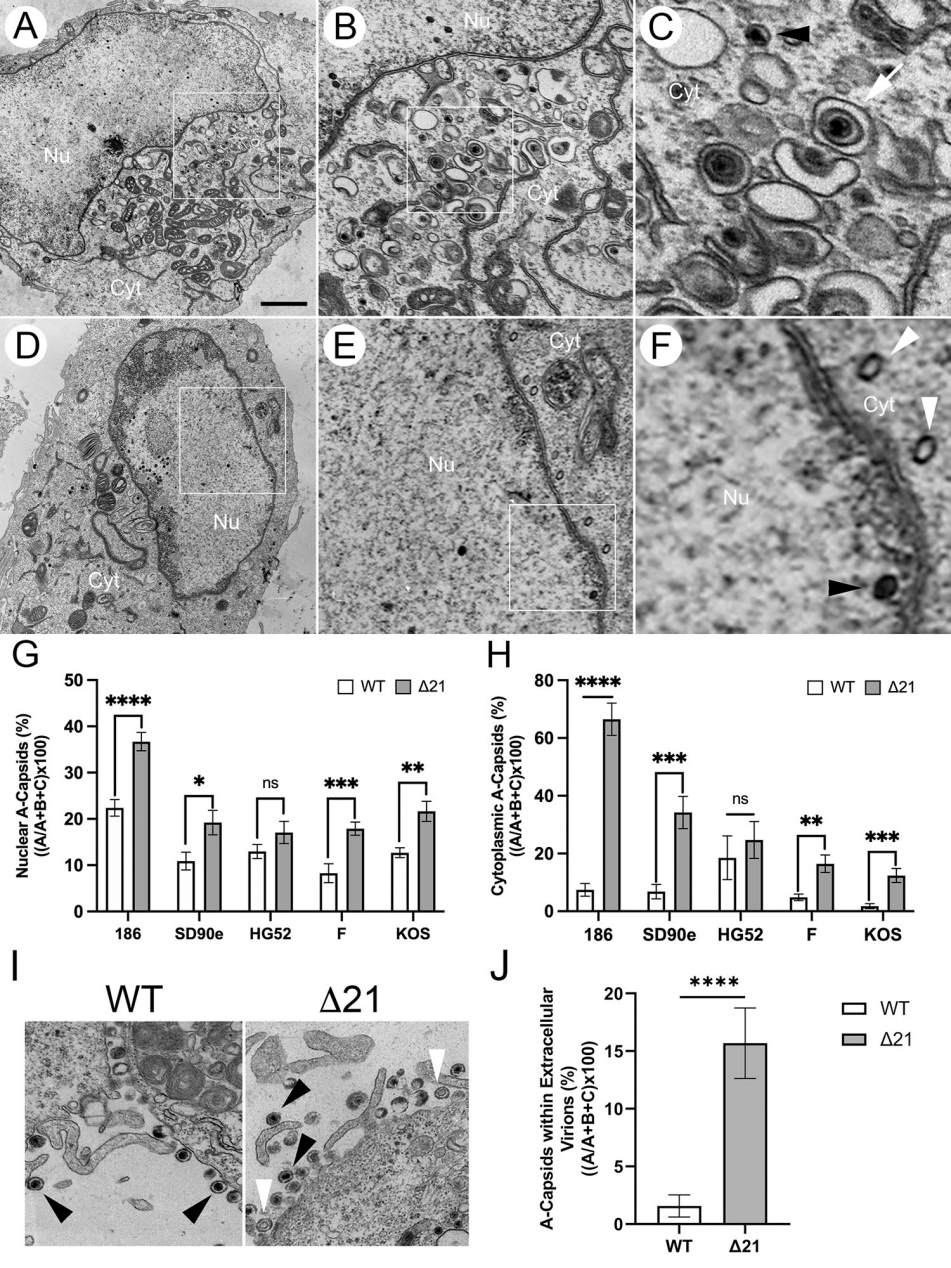
Nuclear and cytoplasmic A-capsid accumulation in HSV WT and Δ21 strains. Vero cells were infected with either an HSV-1 or HSV-2 WT virus or its corresponding Δ21 mutant at an MOI of 3. At 18 hpi, samples were fixed and prepared for TEM. Micrographs for each strain of HSV-1 and HSV-2 were obtained and WT and Δ21 capsids in the nucleus and cytoplasm of infected cells were quantified. **A-F)** Example micrographs of HSV-2 SD90e WT **(A, B,** and **C)** compared to its Δ21 mutant **(D, E,** and **F)**. As depicted in the Δ21 panels (**E** and **F**), there is an abundance of A-capsids (white arrowheads) compared to the WT strain (**B** and **C**). Black arrowheads indicate C-capsids and white arrows indicate enveloped C-capsids. The 2μm scale bar applies to panels **A** and **D**. Panels **B** and **E** are three-fold magnified images of panels **A** and **D**. Panels **C** and **F** are three-fold magnified images of panels **B** and **E**. **G)** The quantification of nuclear A-capsids in cells infected with HSV WT and Δ21 strains are depicted (n = 9–12 micrographs per condition). **H)** The quantification of cytoplasmic A-capsids in cells infected with HSV WT and Δ21 strains are depicted (n = 11–14 micrographs per condition). **I)** Example micrographs of HSV-1 F WT and Δ21 extracellular virions. As depicted in the Δ21 mutant micrograph, there is an abundance of A-capsids within Δ21 extracellular virions (white arrowheads) compared to the WT strain which has mostly C-capsids in extracellular virions (black arrowheads). **J)** The quantification of A-capsids within HSV-1 F WT and Δ21 extracellular virions are depicted (n = 11–14 micrographs per condition). Unpaired t-tests were conducted between Δ21 mutants and WT viruses. *, **, ***, and **** represent p ≤ 0.05, p ≤ 0.01, p ≤ 0.001 and p ≤ 0.0001, respectively. ns = not significant.

To quantify the numbers of nuclear A-capsids in Δ21 infected cells, multiple WT and Δ21 mutant strains derived from HSV-1 and HSV-2 were used to infect Vero cells for transmission electron microscopy (TEM) analysis (Fig 1A–1F). Cells infected with HSV-1 and HSV-2 Δ21 mutants had higher percentages of nuclear A-capsids compared to WT infected cells (Fig 1G). Unpaired t-tests were conducted for HSV-2 (186, SD90e, HG52) and HSV-1 (F, KOS) strains to compare the percentage of nuclear A-capsids in Δ21 infections to WT infections. The Δ21 mutants of HSV-2 186 and SD90e, as well as HSV-1 F and KOS, had significantly higher percentages of nuclear A-capsids present in infected cells compared to corresponding WT infections. There was no significant difference (p = 0.0846) between the HSV-2 HG52 Δ21 mutant and WT virus, despite a higher percentage of nuclear A-capsids in Δ21 infected cells.

TEM micrographs were also used to quantify the number of A-capsids in the cytoplasm of infected cells. The percentage of cytoplasmic A-capsids in cells infected with HSV-2 (186, SD90e, HG52) and HSV-1 (F, KOS) WT viruses, along with their corresponding Δ21 mutants, were determined (Fig 1H). The Δ21 mutants of HSV-2 186 and SD90e, as well as HSV-1 F and KOS, had significantly higher percentages of A-capsids present in the cytoplasm compared to the WT infections. There was no significant difference (p = 0.2701) between the HSV-2 HG52 Δ21 mutant and WT virus, despite a higher percentage of cytoplasmic A-capsids in Δ21 infected cells.

Additionally, TEM micrographs were used to quantify the number of A-capsids packaged within extracellular virions. Previous studies examining PRV mutants that cause rupture of the nuclear envelope and leakage of A-, B-, and C-capsids into the cytoplasm found that these mutants efficiently incorporated A- and B-capsids into extracellular virions [43,44]. This suggests that during PRV infection, C-capsids are not preferentially selected for secondary envelopment. We quantified the percentage of A-capsids within HSV-1 F WT and Δ21 extracellular virions associated with the plasma membrane (Fig 1I). Interestingly, similar to PRV, A-capsids were observed within WT and Δ21 extracellular virions and were observed more frequently packaged into Δ21 extracellular virions compared to the WT strain (Fig 1J).

These data demonstrate that cells infected with Δ21 mutants from all HSV-1 strains tested and two out of three HSV-2 strains have significantly more nuclear and cytoplasmic A-capsids compared to WT infected cells. Moreover, the data suggest that HSV-1 WT and Δ21 cytoplasmic A-capsids are readily enveloped and that more A-capsids are found in extracellular virions in the absence of pUL21.

### Nuclear integrity remains intact in Δ21 infected cells

**Fig 2:**
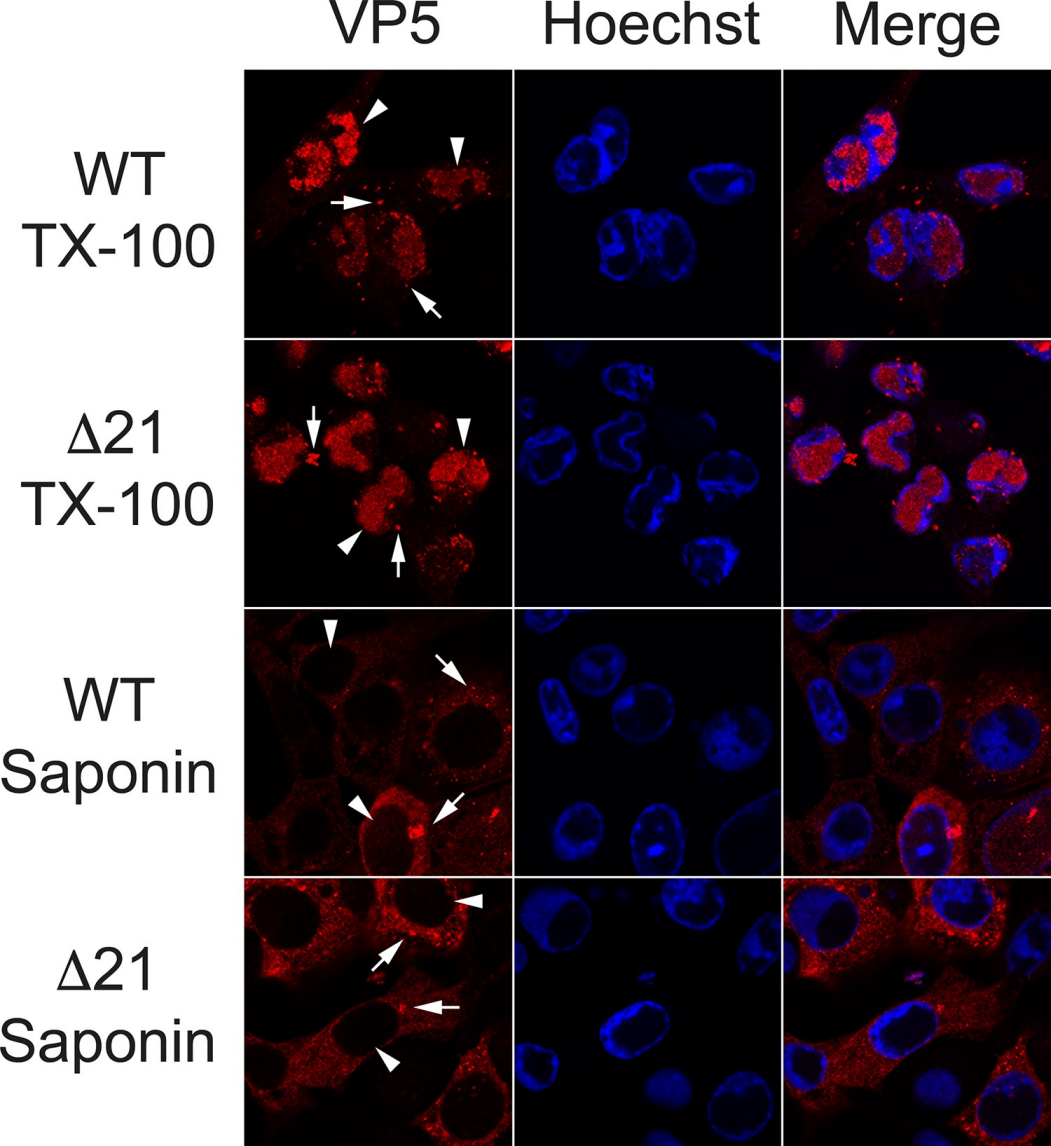
Nuclear integrity remains intact in ΔUL21 infected cells. Vero cells were infected with HSV-1 KOS WT or Δ21 viruses at an MOI of 0.1. At 18 hpi, cells were fixed, permeabilized with triton X-100 (TX-100) or saponin and stained for the major capsid protein VP5. WT and Δ21 infected cells permeabilized with TX-100 have VP5 capsid signal in the nucleus and the cytoplasm. WT and Δ21 infected cells permeabilized with saponin have cytoplasmic VP5 capsid signal (arrows), but no nuclear VP5 capsid signal (arrowheads) indicating that the nuclear envelope is intact in infected cells. Images are representative for three biological replicates per condition.

The data described thus far demonstrated that, in the absence of pUL21, there were more nuclear, cytoplasmic, and extracellular A-capsids, suggesting that genome retention within capsids was impaired. However, if nuclear envelope integrity was compromised during Δ21 infection, passive leakage of A-capsids from the nucleoplasm into the cytoplasm may occur. To evaluate nuclear integrity during Δ21 infection, Vero cells were infected with HSV-1 KOS WT or Δ21. At 18 hpi, cells were fixed, permeabilized with saponin or triton X-100 (TX-100) and stained for the major capsid protein VP5. TX-100 permeabilizes both the plasma membrane and nuclear envelope, while saponin permeabilizes the plasma membrane but not the nuclear envelope. Thus, if the nuclear envelope is intact, saponin permeabilized cells will display only cytoplasmic VP5 signal as the antibodies used for VP5 detection are unable to access the nucleoplasm. Cytoplasmic and nuclear VP5 staining was observed in WT and Δ21 infected cells permeabilized with TX-100, while only cytoplasmic VP5 signal was observed in WT and Δ21 infected cells permeabilized with saponin (Fig 2). These data indicate that the integrity of the nuclear envelope remains intact in WT and Δ21 infected cells and strongly suggest that A-capsid accumulation in the cytoplasm of Δ21 infected cells was not due to the loss of nuclear envelope integrity during infection. In further support of this conclusion, we failed to identify B-capsid accumulation in the cytoplasm of Δ21 infected cells (Fig 1A–1F), arguing against gross perturbations of nuclear envelope barrier function in these cells.

### Preferential selection of C-capsids for nuclear egress is operational in the absence of pUL21

**Fig 3:**
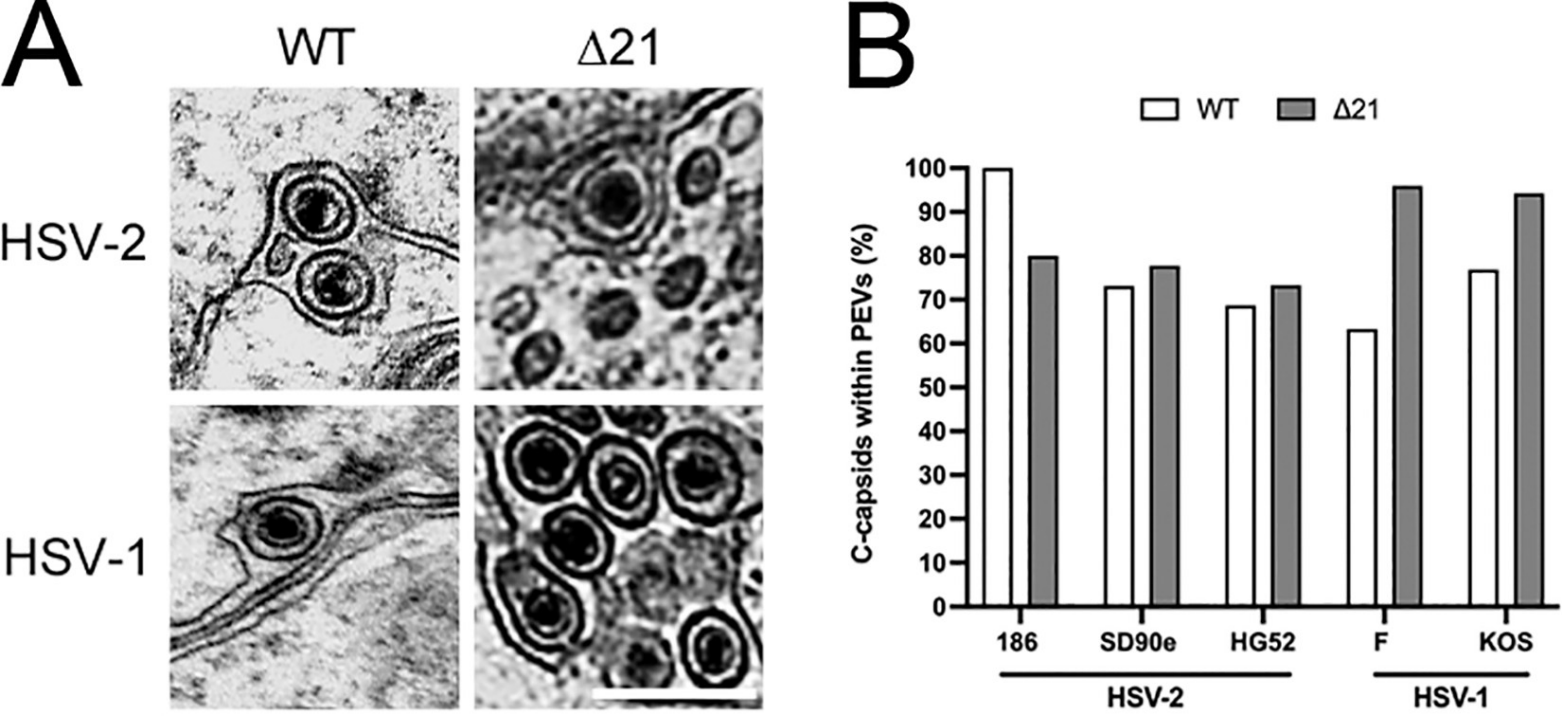
Perinuclear C-capsid abundance in HSV WT and Δ21 infected cells. Vero cells were infected with HSV-1 or HSV-2 WT strains or their corresponding Δ21 mutants at an MOI of 3 for 18 h. Cells were then fixed and prepared for TEM. Micrographs for each strain of HSV-1 and HSV-2 were obtained to quantify WT and Δ21 capsids within PEVs. **A)** Examples of micrographs containing PEVs derived from HSV-2 SD90e WT and its Δ21 mutant and HSV-1 F WT and its Δ21 mutant. The scale bar shown is 500nm and applies to all panels. **B)** The quantification of C-capsids within PEVs from cells infected with HSV WT and Δ21 strains (n = 11–27 micrographs per condition). A total of 7–158 PEVs were analyzed per condition. Statistical analysis was not possible due to PEVs being a transient assembly intermediate and not consistently present within each micrograph.

Another possible explanation for the increased numbers of A-capsids in the cytoplasm of Δ21 infected cells is that there is a breakdown in C-capsid selectivity during nuclear egress such that A-capsids acquire primary envelopes efficiently, enabling their delivery to the cytoplasm. To address this, we quantified primary enveloped virions (PEVs) by TEM (Fig 3A). If C-capsid selectivity was functional in the absence of pUL21, C-capsids should be preferentially found within PEVs. Capsids inside PEVs were scored as A-capsids, B-capsids, C-capsids, or unidentified (i.e., capsids that could not be unambiguously identified as A, B, or C). Minimal differences were seen in the percentage of C-capsids within PEVs when comparing HSV-1 (F, KOS) and HSV-2 (186, SD90e, HG52) Δ21 mutants to their corresponding WT viruses (Fig 3B). Indeed, most Δ21 strains had more C-capsids within PEVs. This may be due to the accumulation of PEVs in the perinuclear space, previously observed in the absence of pUL21 [30]. Nevertheless, these analyses suggest that the selectivity of C-capsids for nuclear egress remained functional in the absence of pUL21 and that enhanced selection of A-capsids for primary envelopment is unlikely to be responsible for the accumulation of A-capsids in the cytoplasm.

### Viral genome retention within capsids is compromised in the absence of pUL21

**Fig 4:**
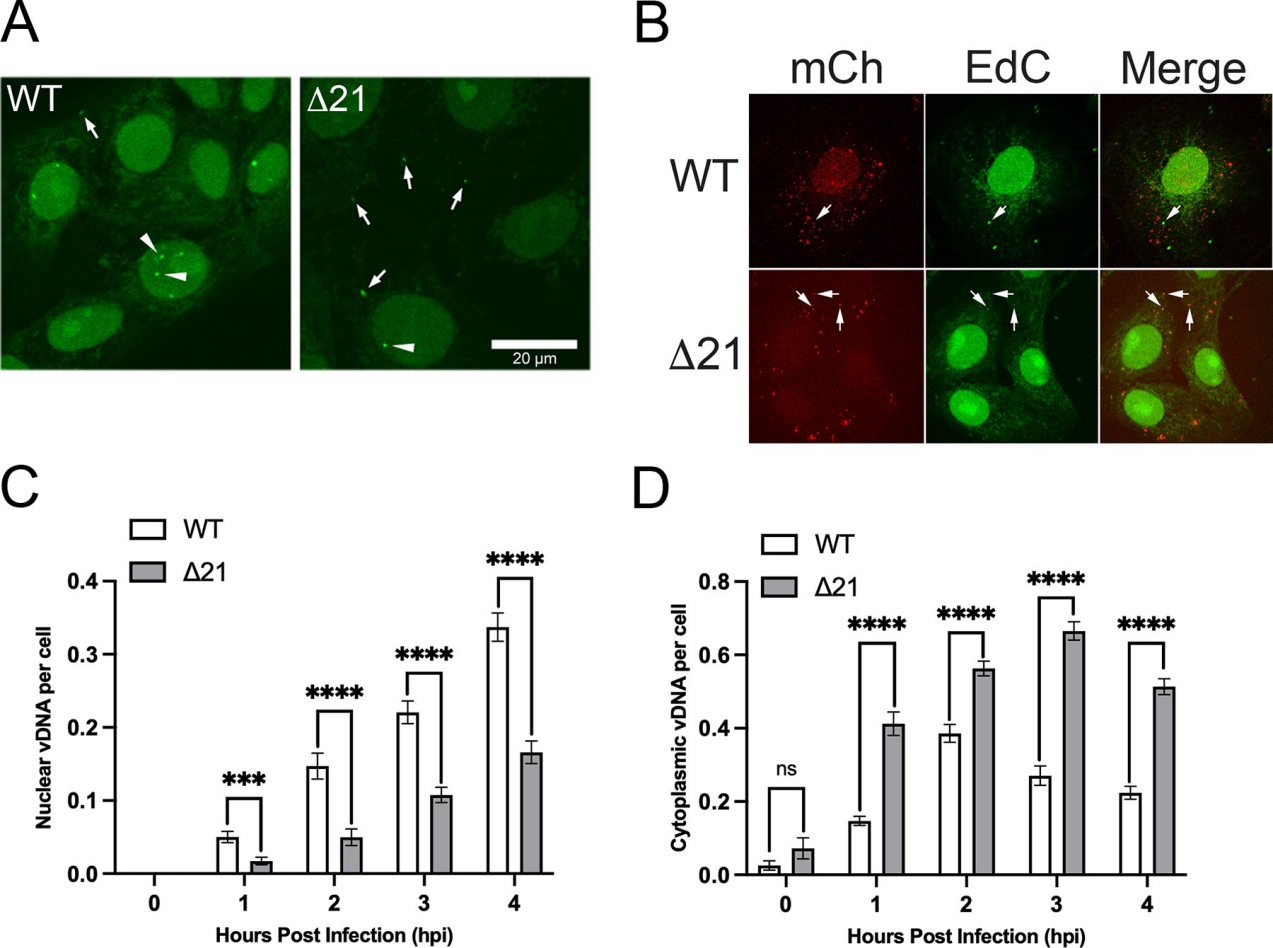
Comparison of genome retention of HSV-1 KOS WT and Δ21 capsids during early infection. Vero cells were infected at an MOI of 0.1 with HSV-1 KOS WT or Δ21 viruses containing EdC incorporated into their genomes. Infected cells were fixed at early times after infection (0–4 hpi) and genomes ejected from capsids were labeled using click-chemistry. **A)** Representative images of WT and Δ21 infected cells at 4 hpi with ejected viral genomes labeled using click-chemistry. The scale bar is 20μm. Labelled genomes were identified as intense puncta seen in the cytoplasm (arrows) and nuclei of infected cells (arrowheads). Puncta were quantified and their numbers divided by the total number of cells to determine nuclear and cytoplasmic genomes per cell. **B)** Control experiment using EdC labeled HSV-2 186 WT or Δ21 that produces mCh labeled nucleocapsids (MOI 3) showing that click-chemistry does not label genomes within capsids at 2 hpi. Arrows point to EdC labelled puncta that abut mCh labelled capsids. Note that the majority of mCh labelled capsids do not co-localize with an EdC signal. **C)** The quantification of nuclear viral genomes (vDNA) per cell over 0–4 hpi is shown (n = 21 images per timepoint and condition). **D)** The quantification of cytoplasmic genomes (vDNA) per cell over 0–4 hpi is shown (n = 21 images per timepoint and condition). Unpaired t-tests were performed between Δ21 mutants and WT viruses for three biological replicates. *** and **** represent p ≤ 0.001 and p ≤ 0.0001, respectively.

To evaluate WT and Δ21 genome retention within capsids, we examined genome ejection during the early stages of infection using click-chemistry. Early in infection, after fusion of the virion envelope with a cellular membrane, capsids containing viral genomes are transported to nuclear pore complexes through which genomes are deposited into the nucleus. However, if capsids fail to retain their genomes, they are prematurely ejected into the cytoplasm, which can be quantified. HSV-1 KOS WT and Δ21 strains were propagated in the presence of the nucleoside analog, EdC, which was incorporated into the genomes of progeny virions. Viruses containing EdC-labelled genomes were then used to infect Vero cells growing on glass-bottom dishes. Cells were fixed at intervals between 0 and 4 hpi and click-chemistry was performed to fluorescently label EdC within viral genomes that had been ejected from capsids (Fig 4A). Importantly, EdC-labelled genomes that were contained within capsids were not fluorescently labelled by the click reaction (Fig 4B). Fluorescent puncta, representing individual viral genomes, were quantified to determine the number of nuclear (Fig 4C) and cytoplasmic (Fig 4D) viral genomes released from capsids per cell, over time. Δ21 infected cells had significantly fewer viral genomes delivered to nuclei between 1 and 4 hpi compared to WT (Fig 4C). Additionally, Δ21 infected cells had significantly more non-encapsidated viral genomes in the cytoplasm between 1 and 4 hpi compared to WT (Fig 4D). These findings indicate that HSV-1 KOS Δ21 capsids have impaired viral genome retention, and may explain the abundance of A-capsids observed in the cytoplasm of Δ21 infected cells late in infection.

**Fig 5:**
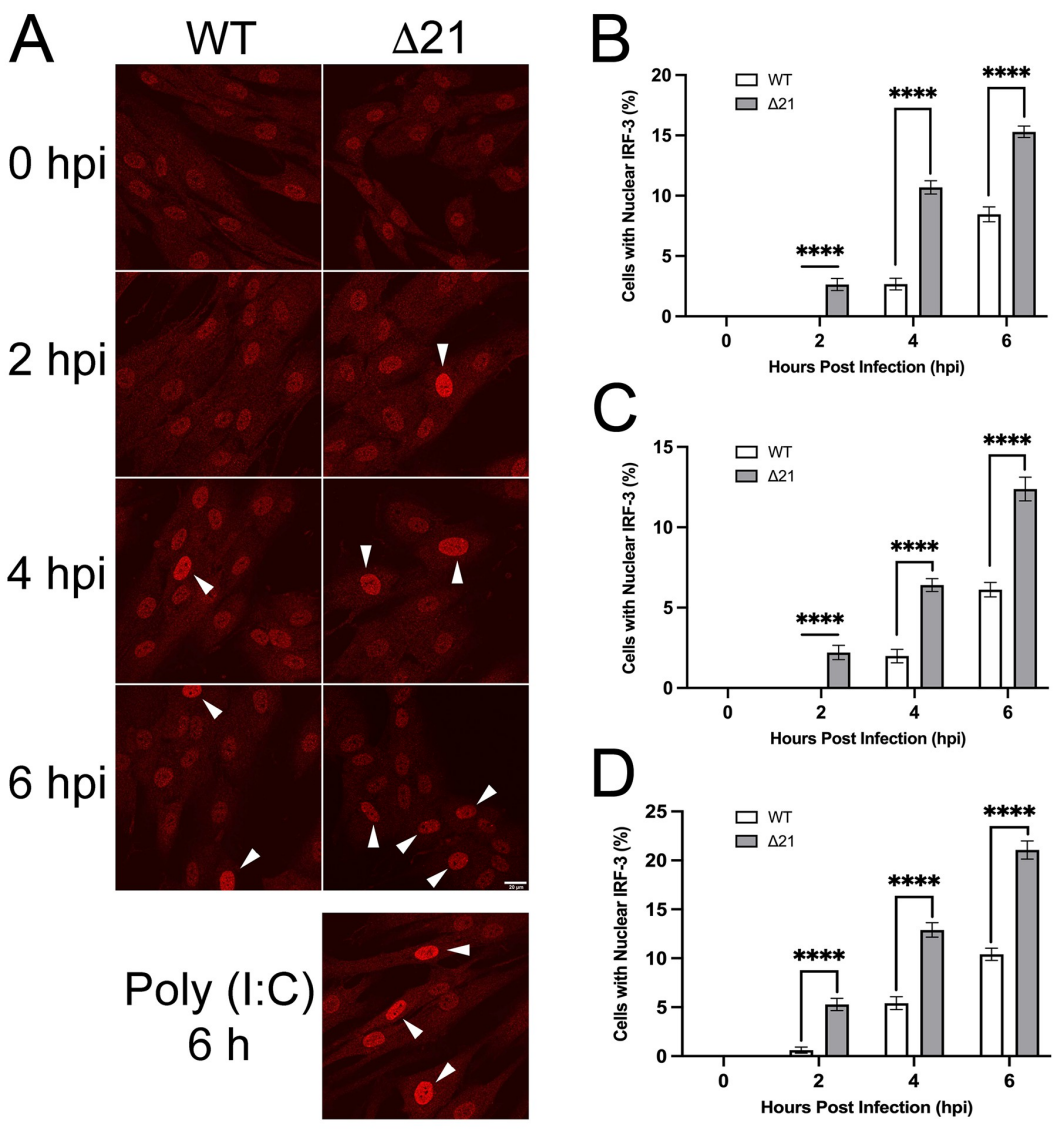
Examination of IRF-3 nuclear localization during early stages of infection with HSV WT and Δ21 strains. T12 cells were infected at an MOI of 3 and after inoculation for one-hour on ice, cells were incubated in medium containing 50 μg/ml of cycloheximide to inhibit protein synthesis. Infected cells were fixed at 0, 2, 4 and 6 hpi, stained for IRF-3 and imaged by confocal microscopy. **A)** Representative images of HSV-1 KOS WT and Δ21 infected cells. IRF-3 localization at 0, 2, 4, and 6 hpi. Nuclear IRF-3 is indicated by the white arrowheads. Representative image of T12 cells transfected for 6 hours with poly I:C is shown as a positive control. (n = 33–37 images per timepoint and condition). Scale bar is 20μm and applies to all panels. **B)** Quantification of nuclear IRF-3 in cells infected with HSV-1 KOS WT and Δ21 strains (n = 33–36 images per timepoint and condition). **C)** Quantification of nuclear IRF-3 in cells infected with HSV-1 F WT and Δ21 strains (n = 36 images per timepoint and condition). **D)** Quantification of nuclear IRF-3 in cells infected with HSV-2 SD90e WT and Δ21 strains (n = 36 images per timepoint and condition). Unpaired t-tests were performed to compare the percentage of cells with nuclear IRF-3 for each timepoint in Δ21 mutants with WT virus for each strain, from three biological replicates. **** represents p ≤ 0.0001.

As a complementary approach to analyze premature ejection of viral genomes in other HSV-1 and HSV-2 Δ21 mutants, activation of the cGAS/STING pathway was examined. When viral genomes are prematurely ejected into the cytoplasm, they can be detected by components of the cGAS/STING pathway leading to interferon regulatory factor 3 (IRF-3) phosphorylation in the cytoplasm and its subsequent translocation to the nucleus [46,47]. Thus, nuclear translocation of IRF-3 can be used as a surrogate measure of premature ejection of viral genomes into the cytoplasm of infected cells [47,48]. Due to the severe growth defect of HSV-2 186 Δ21 virus [42], this strain was not investigated in these experiments. T12 cells, life extended human fibroblasts, were used for these experiments because, unlike Vero cells, they can produce interferon and thus have intact IRF-3 signalling pathways. T12 cells were infected with WT or Δ21 viruses at an MOI of 3. Cells with nuclear IRF-3 were quantified in WT and Δ21 infected cells by confocal microscopy (Fig 5A). At 2, 4, and 6 hpi, HSV-1 F and KOS **Δ**21 infections had significantly more cells with nuclear IRF-3 compared to their respective WT infections (Fig 5B and 5C). Additionally, during HSV-1 F and KOS Δ21 infections, translocation of IRF-3 to infected nuclei occurred 2 hours earlier than their respective WT infections. At 2, 4, and 6 hpi, HSV-2 SD90e **Δ**21 infection also displayed significantly more cells with nuclear IRF-3 compared to WT (Fig 5D). It should be noted that several different pattern recognition receptors which signal through IRF-3 can be activated upon HSV infection and detect HSV virions [49]. Despite this limitation, collectively our results suggest that Δ21 mutants prematurely eject more viral genomes into the cytoplasm (Figs 4D and 5A–5D) and deliver fewer genomes to the nuclei of infected cells (Fig 4C).

### The composition of nuclear capsids is altered in the absence of pUL21

Previous studies examining viruses mutated for capsid and tegument proteins have reported changes in capsid and tegument composition [7,14,50–53]. To understand the mechanism by which Δ21 capsids have impaired genome retention we investigated capsid composition by mass spectrometry. After the exclusion of common contaminants, a total of 265 unique cellular and viral proteins were identified in HSV-1 KOS WT and Δ21 nuclear A-, B-, and C-capsid preparations (S1 Table). Variations between capsid abundance in different biological replicates as well as between WT and Δ21 strains were normalized using the total spectrum count abundance of the major capsid protein, VP5, which is found in exactly 955 copies per capsid [54]. Next, the percent change of each identified protein was determined and averaged over the three biological replicates for Δ21 A-, B-, and C-capsids compared to WT A-, B-, and C-capsids. We defined a significant protein change as being greater than a 20% increase, or decrease, in spectrum count abundance. Of the 265 identified proteins, the tegument protein pUL16 was increased by 9.68%, 96.52%, and 161.75% on Δ21 A-, B-, and C-capsids, respectively, compared to WT capsids. pUL16 is a known binding partner of pUL21 and, interestingly, its association with capsids is thought to occur only in the cytoplasm of infected cells [35,55]. The CVSC is known to be vital for capsid stability and genome retention [14]. The amount of the CVSC proteins, pUL17 and pUL25, were similar when comparing Δ21 capsids to WT capsids. Interestingly, the CVSC protein pUL36 was decreased by 37.19% on Δ21 A-capsids compared to WT A-capsids (S1 Table). Unlike pUL25, pUL36 has not been implicated in genome retention and pUL36 mutants have a similar amount of nuclear A-, B-, and C-capsids compared to WT infected nuclei [14]. Given this, it is unlikely that alterations in pUL36 levels are impairing genome retention within capsids in the absence of pUL21. Furthermore, no cellular proteins that met our defined criteria stood out as candidates for impairing capsid genome retention in the absence of pUL21. Thus, we next examined if pUL16 was increased on a variety of HSV-1 and HSV-2 Δ21 capsids by western blot analysis.

### Increased levels of pUL16 are associated with HSV-1 and HSV-2 nuclear capsids in the absence of pUL21

**Fig 6:**
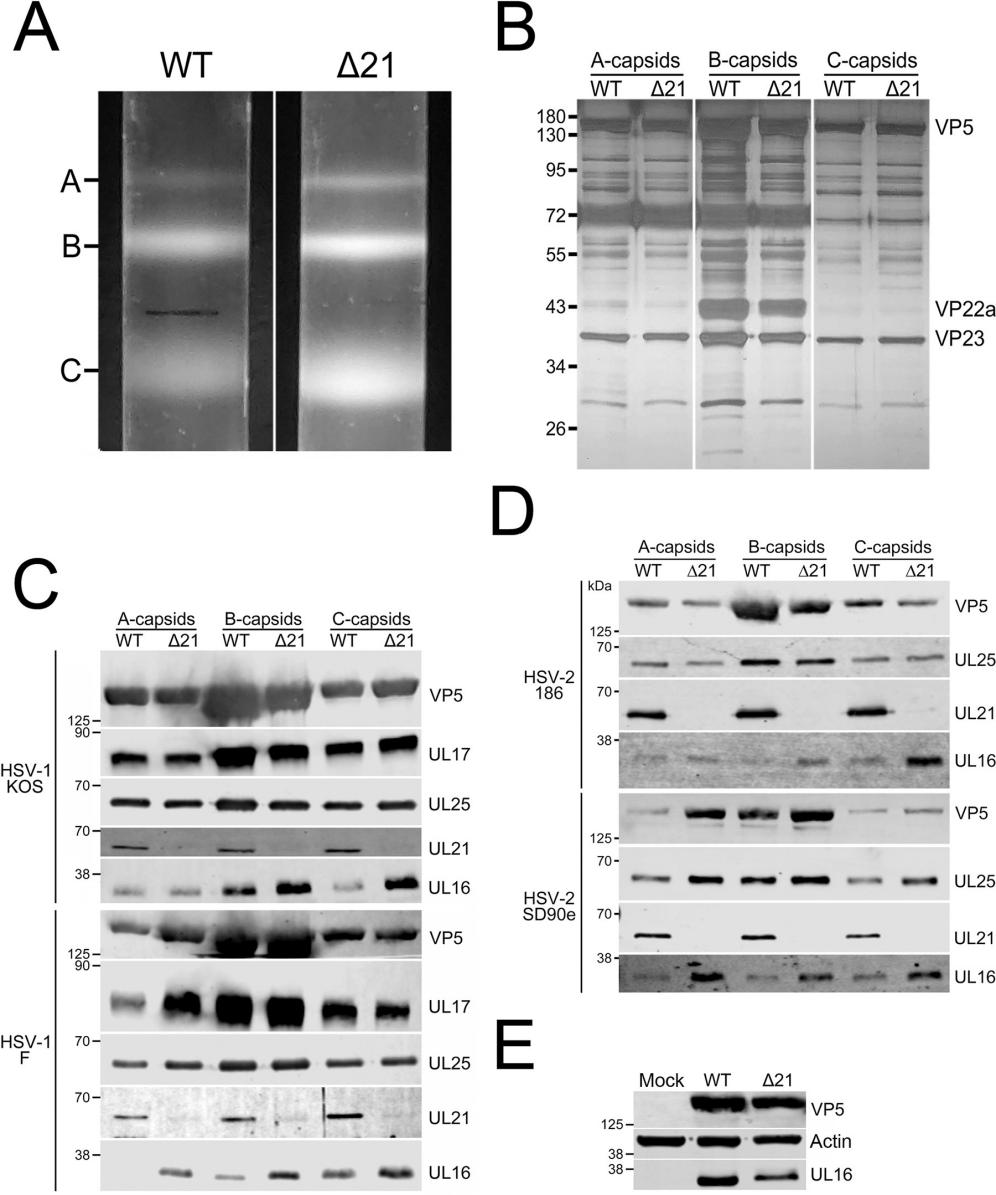
Isolation and analysis of HSV WT and Δ21 nuclear A-, B-, and C-capsids. Nuclear capsids were isolated from Vero cells infected with HSV-1 or HSV-2 WT and corresponding Δ21 strains. **A)** Light scattering bands observed in 20–50% sucrose gradients used to resolve A-, B- and C-capsids isolated from cells infected with WT and Δ21 HSV-1 KOS. **B)** Nuclear capsid-containing gradient fractions from WT and Δ21 HSV-1 KOS infected cells were electrophoresed through an SDS-PAGE gel and silver-stained to identify proteins associated with A-, B-, and C-capsids. VP5 and VP23 can be clearly seen in all samples, while VP22a is only seen in WT and Δ21 B-capsid fraction samples. No obvious differences were observed between WT and Δ21 A-, B-, and C-capsids**. C** and **D)** Western blot analysis of HSV-1 (KOS, F) (three and two biological replicates, respectively) and HSV-2 (186, SD90e) (one and two biological replicates, respectively). WT and Δ21 A-, B-, and C-capsid fractions probed with antisera, indicated on the right side of each panel. Migration positions of molecular weight markers (kDa) are indicated on the left side of each panel. **E)** Vero cells were mock-infected or infected with HSV-1 KOS WT or Δ21 strains (MOI 3). At 18 hpi, cell lysates were analyzed by western blotting using the antisera indicated on the right side of each panel. The migration position of molecular weight markers (kDa) are indicated on the left side of each panel. Three biological replicates were examined.

Nuclear capsids were isolated from infected cells by ultracentrifugation through 20–50% sucrose gradients to resolve A-, B-, and C-capsids (Fig 6A). No apparent differences were seen when comparing the light-scattering bands of Δ21 A-, B-, and C-capsids to the light-scattering bands of WT A-, B-, and C-capsids. Sucrose gradients were fractionated, and capsid-containing fractions were subjected to SDS-PAGE followed by silver-staining (Fig 6B). No obvious differences in protein profiles were seen between WT and Δ21 capsids.

WT and Δ21 capsids were then analyzed by western blotting using antisera reactive against VP5, pUL17, pUL25, pUL21, and pUL16. Blots were imaged using a LI-COR CLx imager to assess differences in capsid protein composition. WT and Δ21 capsid loading levels were normalized using the signal obtained from the major capsid protein VP5. While there were modest fluctuations in the amounts of pUL17 and pUL25 present in A-, B- and C-capsids from WT and Δ21 HSV-1 and HSV-2 strains, none of these fluctuations were consistent between strains (Fig 6C and 6D and Table 1). For example, while A-capsids from HSV-1 F Δ21 had more pUL17 than the parental WT strain, this was not the case for HSV-1 KOS (Fig 6C). Likewise, while C-capsids from HSV-2 SD90e Δ21 had less pUL25 than the parental WT strain (Fig 6D), this was not the case for the other HSV-2 and HSV-1 strains investigated. The only difference in capsid composition consistently observed between all HSV-1 and HSV-2 strains analyzed was an increase in pUL16 associated with Δ21 capsids in comparison to their WT counterparts.

**Table 1:** Qualitative analysis of capsid associated proteins.

Strain ^*a*^	Protein	Number of Biological Replicates	Average Protein Change (%) (Δ21 compared to WT) ^*b*^		
			A-capsid	B-capsid	C-capsid
HSV-1 KOS	pUL17	3	-19 ± 30	1 ± 6	0 ± 21
	pUL25		-6 ± 30	7 ± 19	-2 ± 9
	pUL16		80 ± 33	169 ± 89	197 ± 150
HSV-1 F	pUL17	2	86 ± 11	4 ± 3	8 ± 17
	pUL25		17 ± 17	-12 ± 8	39 ± 5
	pUL16		189 ± 8	134 ± 13	83 ± 26
HSV-2 186	pUL25	1	4	12	40
	pUL16		109	275	663
HSV-2 SD90e	pUL25	2	-12	-37	-30
	pUL16		83 ± 74	71 ± 45	31 ± 2

^*a*^ Quantification data presented is from western blots in Fig 6C and 6D.

^*b*^ The mean ± standard error of the mean is shown. For samples with only one biological replicate, only the mean is shown.

A trivial explanation for these findings is that increased pUL16 expression in the absence of pUL21 led to enhanced pUL16 association with capsids. To eliminate this possibility, we examined pUL16 expression levels in Δ21 infected cells (Fig 6E). WT and Δ21 infected cell lysates were normalized using VP5 signal intensity. Δ21 infected cells had 38% less pUL16 compared to WT infected cells. This suggests that the increased association of pUL16 with Δ21 nuclear capsids was not due to elevated expression levels of pUL16 in Δ21 infected cells.

### Deletion of UL16 from HSV-1 KOS Δ21 decreased the abundance of nuclear A-capsids in infected cells

**Fig 7:**
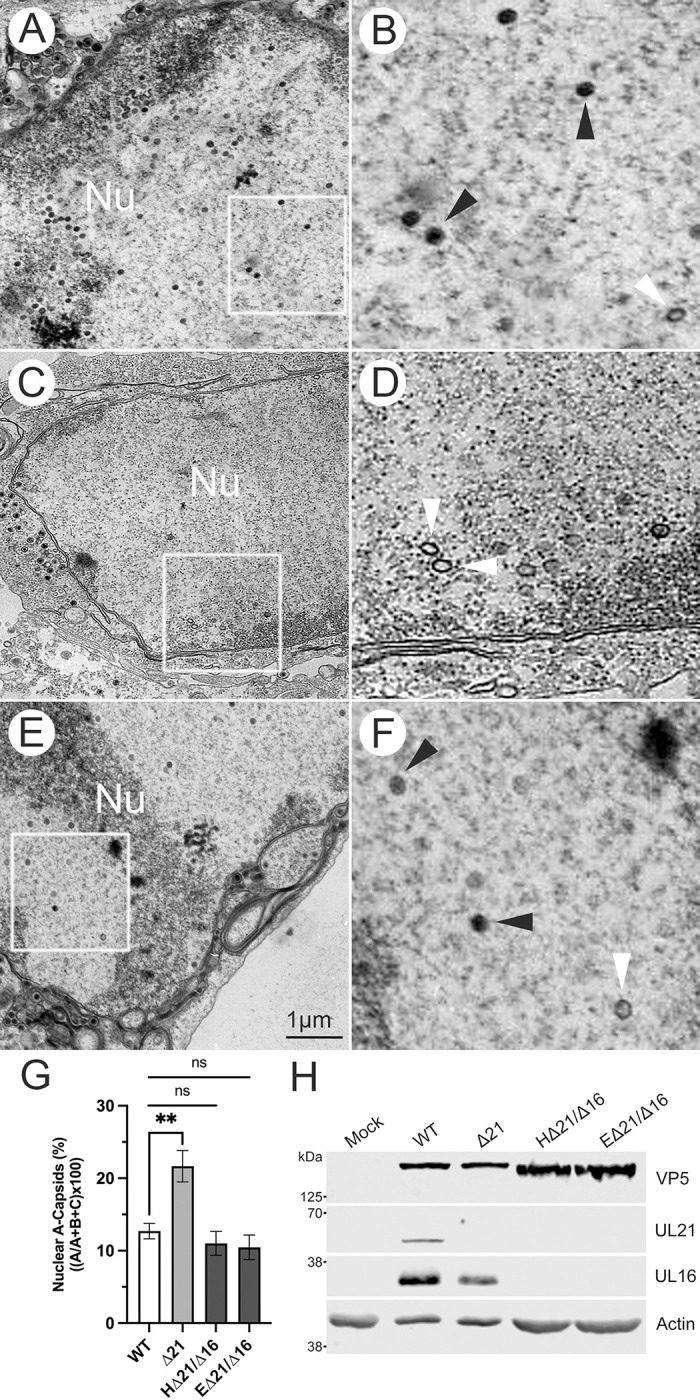
Characterization of capsids produced by Δ21/Δ16 double mutants. **A-F)** Vero cells were infected with either HSV-1 KOS WT **(A** and **B)**, Δ21 **(C** and **D)**, or EΔ21/Δ16 strains **(E** and **F)** at an MOI of 3 for 18 hpi and examined by TEM. The 1μm scale bar applies to panels **A**, **C**, and **E**. White arrowheads indicate nuclear A-capsids and panels **B**, **D**, and **F** are three-fold magnified images of panels **A**, **C**, and **E**, respectively. **G)** Quantification of nuclear A-capsids in cells infected with the indicated HSV-1 KOS derived strains (n = 11–14 micrographs per condition). WT and Δ21 data used for this analysis are the same as those included in Fig 1G. A one-way ANOVA with Tukey’s HSD Test for multiple comparisons were performed between all viruses. ** p ≤ 0.01. The difference in the percentage of nuclear A-capsid was not significant when comparing HΔ21/Δ16 (p = 0.886) and EΔ21/Δ16 (p = 0.781) mutants to WT. **H)** Western blot analysis of lysates prepared from Vero cells infected with the indicated strains at an MOI of 3 at 18 hpi. Antisera used are indicated on the right side of each panel. The migration position of molecular weight markers (kDa) are indicated on the left side of each panel.

We reasoned that if the increased association of pUL16 with Δ21 nuclear capsids was impairing genome retention, then deletion of UL16 from HSV-1 KOS Δ21 strain would restore genome retention. We used CRISPR/Cas9 mutagenesis to construct two independent HSV-1 KOS Δ21/Δ16 mutant strains (HΔ21/Δ16 and EΔ21/Δ16). These strains were analyzed by TEM and numbers of A-, B- and C-capsids were quantified in the nuclei of infected cells (Fig 7A–7F). Interestingly, limited numbers of cytoplasmic capsids were seen in Δ21/Δ16 infected cells, suggesting that the combined effect of deleting both UL21 and UL16 genes led to decreased nuclear egress. Whilst the Δ21 mutant had roughly twice as many A-capsids in the nucleus as the parental WT strain, both Δ21/Δ16 double mutants had numbers of A-capsids that were indistinguishable from the WT strain. A one-way ANOVA analysis revealed a significant difference in the percentage of nuclear A-capsids between at least two viruses (F(3, 46) = 8.95, p<0.001). Tukey’s HSD Test for multiple comparisons found that the mean percentage of nuclear A-capsids was significantly different between WT and Δ21 (p<0.01, 95% C.I. = [-15.55, -2.35]), Δ21 and HΔ21/Δ16 (p<0.001, 95% C.I. = [-17.01, -4.27]), as well as Δ21 and EΔ21/Δ16 (p<0.001, 95% C.I. = [-17.67, -4.72]). There was no significant difference in the percentage of nuclear A-capsids between WT and HΔ21/Δ16 (p = 0.886), nor WT and EΔ21/Δ16 (p = 0.781) (Fig 7G). Additionally, we confirmed that both HΔ21/Δ16 and EΔ21/Δ16 did not express pUL21 and pUL16 in infected cells (Fig 7H). These findings demonstrate that the absence of both pUL16 and pUL21 restored the ability of capsids to retain genomes at WT levels. These data, coupled with our findings that HSV-1 and HSV-2 Δ21 mutants have more capsid-associated pUL16 compared to WT capsids, suggest that increased and/or premature association of pUL16 with nuclear capsids in the absence of pUL21 impairs capsid genome retention.

## Discussion

In this study we have defined the mechanism by which A-capsids accumulate in cells infected with multiple HSV-1 and HSV-2 UL21 deletion mutants. We found that A-capsids accumulated in the cytoplasm and nuclei of Δ21 infected cells and that capsids lacking genomes were more commonly incorporated into Δ21 extracellular virions compared to WT viruses (Fig 1). Interestingly, despite HSV-2 strain HG52 having more nuclear and cytoplasmic A-capsids present in Δ21 infected cells compared to WT infected cells, this was the only strain where the differences between WT and Δ21 were not significant (Fig 1). Given that the pUL21 proteins from HSV-2 186, SD90e and HG52 are identical, it was surprising that strain HG52 was an outlier. When comparing the proportion of nuclear and cytoplasmic A-capsids from each WT infection, HG52 infected cells had the second highest percentage of nuclear A-capsids (Fig 1G) and the highest percentage of cytoplasmic A-capsids (Fig 1H). This observation suggests that genome packaging, or the formation of HG52 capsids, is inherently defective relative to the other strains investigated and that the absence of pUL21 might not have as great an impact on A-capsid accumulation as it would in other strains. It may be that HG52 has polymorphisms outside the UL21 locus that influence capsid formation or genome retention/packaging. It is perhaps noteworthy that while pUL16 is identical in HSV-2 strains 186 and SD90e there is a stretch of five amino acids between residues 182 and 188 that differ in HG52 pUL16. It may be that HG52 pUL16 has a propensity to prematurely associate with nuclear capsids in the presence of pUL21; however, this needs to be addressed experimentally.

**Fig 8:**
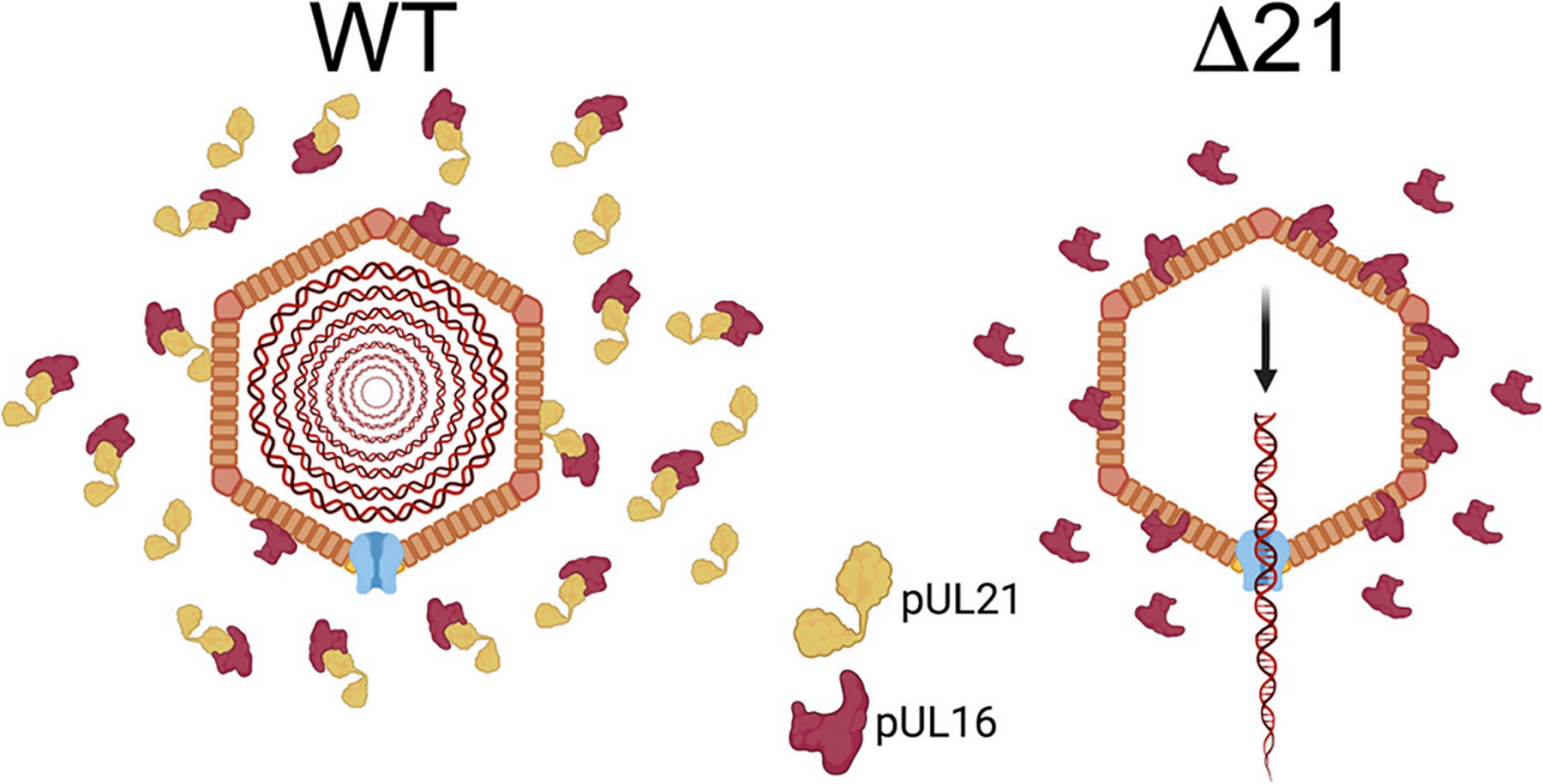
Possible mechanism accounting for impaired viral genome retention in the absence of pUL21. In the nuclei of cells infected with WT strains, sequestration of pUL16 by pUL21 prevents pUL16 from associating with nucleocapsids. However, in the absence of pUL21, pUL16 more readily associates with nucleocapsids and hinders genome retention within Δ21 capsids. The figure suggests that viral genomic DNA vacates Δ21 capsids via the portal, however, it may be that genomes escape these capsids at sites other than the portal. Figure created with BioRender.com.

Our experiments indicated that A-capsid accumulation in the cytoplasm of Δ21 infected cells was not due to nuclear envelope rupture (Fig 2) or a breakdown in the preferential selection of C-capsids for nuclear egress (Fig 3). Rather, the data suggest that the ability of capsids to retain viral genomes is compromised in the absence of pUL21 (Figs 4 and 5). Our analysis of capsid genome retention was undertaken during virus entry. An important consideration, however, is that capsid stability during entry may differ during egress, which potentially impacts the interpretation of our findings. Because the level of viral DNA synthesis late in infection produces such a robust EdC signal, such assays preclude our ability to evaluate capsid genome retention during virion egress, which relies on the detection of single genomes that produce comparatively weak EdC signals. Regardless, these findings prompted an analysis of Δ21 capsid composition. Curiously, when nuclear A-, B-, and C-capsids were isolated by ultracentrifugation through sucrose gradients, WT A-capsid bands had a similar intensity to those derived from Δ21 infected nuclei (Fig 6A). Based on the increased proportion of A-capsids seen in the nuclei of Δ21 infected cells by TEM (Fig 1), we might have expected to see a more intense A-capsid band in the Δ21 samples. One explanation for this apparent discrepancy is that the bulk of the A-capsids seen in Δ21 infected cells by TEM are unstable and are not amenable to isolation on sucrose gradients. In support of this concept, cells infected with an HSV-1 pUL25 mutant produce nuclear A-, B-, and C-capsids when assessed by TEM, however, no C-capsid band is present on sucrose gradients of nuclear capsid preparations; presumably, because capsids lacking pUL25 are unable to withstand the capsid isolation procedure [7,14]. Despite this conundrum, our analyses identified more pUL16 on all capsid types isolated from HSV-1 and HSV-2 Δ21 in comparison to WT capsids (Fig 6 and Table 1). Ideally, we would have also performed an analysis of pUL16 co-localization with WT and Δ21 capsids in cells. Unfortunately, this was not possible because our pUL16 antisera does not work well in indirect immunofluorescence assays. Future experiments using cells infected with viruses bearing epitope tagged pUL16 and fluorescent capsids should enable this analysis. In WT infected cells, pUL16 is not thought to associate with capsids until they reach the cytoplasm [55]. As pUL16 has a pan-cellular distribution, this suggests that a mechanism exists to prevent the association of pUL16 with capsids in the nucleus. A simple explanation for these findings is that complex formation between pUL21 and pUL16 in the nucleus prevents pUL16 from associating with capsids (Fig 8).

To investigate this hypothesis, we constructed two independent HSV-1 KOS Δ21/Δ16 mutant strains (HΔ21/Δ16 and EΔ21/Δ16). We could only quantify nuclear A-capsids in HΔ21/Δ16 and EΔ21/Δ16 infected cells as there were limited numbers of cytoplasmic A-capsids in these cells. In HSV-1 mutants lacking pUL21 or pUL16 alone, nuclear egress defects have not been documented [40,56–60]. Thus, it was surprising that in HSV-1 HΔ21/Δ16 and EΔ21/Δ16 infected cells, there were fewer cytoplasmic capsids than in HSV-1 Δ21 and WT infected cells, suggesting these double mutants have a nuclear egress defect. Intriguingly, Benedyk and colleagues have noted that the closest structural homolog to the N-terminal domain of HSV-1 pUL21 is the AlphaFold structural prediction of the pUL16 C-terminal domain [41,61]. It may be that redundant activities within HSV-1 pUL21 and pUL16 can support nuclear egress whereas deletion of both molecules leads to deficiencies in this process. Interestingly, HSV-2 mutants lacking pUL21, or pUL16 alone are deficient in nuclear egress [31,62]. Quantification of nuclear A-capsids in HΔ21/Δ16 and EΔ21/Δ16 infected cells resulted in no significant difference in the proportions of nuclear A-capsids compared to WT infection. However, there were significantly fewer nuclear A-capsids compared to Δ21 infected cells (Fig 7G). These findings indicate that the deletion of UL16 from the HSV-1 KOS Δ21 mutant restored capsid genome retention.

We hypothesize that pUL21 regulates the location and timing of pUL16 addition to capsids (Fig 8) and that during WT infection, pUL16 interaction with pUL21 in the nucleus prevents pUL16 from binding to capsids. How might premature and/or excessive pUL16 association with capsids impact capsid stability? During procapsid formation, the addition of capsomers and triplexes to the scaffolding proteins is regulated and occurs through interactions between pentons and hexons with scaffolding proteins and triplex components [16]. Additionally, during capsid maturation, disulfide bond complexes form between capsid structural components [23–25]. Specifically, disulfide bonds form between the major capsid protein VP5, triplex proteins, CVSC proteins, pUL6 and the tegument protein VP22. Disulfide bonds stabilize HSV capsids and disruption of these bonds can result in impaired capsid stability and genome retention. pUL16 contains 20 cysteines, with 8 of these conserved amongst all herpesviruses [55,63]. Thus, if pUL16 associates with nuclear capsids too early during assembly, it may result in improper disulfide bond formation between capsid components and impair genome retention after packaging. Future examination of disulfide bond complexes formed between capsid proteins in the absence of pUL21 should be conducted to better understand the mechanism(s) by which premature and/or over-addition of pUL16 affects genome retention within capsids.

In summary, this study has addressed a significant knowledge gap by elucidating the role of pUL21 in maintaining capsid stability in multiple HSV-1 and HSV-2 strains. The identification of factors that contribute to capsid stability exposes potential drug targets that can be exploited in the treatment of disease caused by these important human pathogens.

## Materials and methods

### Cells and viruses

African green monkey kidney cells (Vero), life-extended human foreskin fibroblasts (T12), a kind gift from Dr. W. A. Bresnahan (University of Minnesota) [64], human keratinocytes (HaCaT), HaCaT21 and 293T21 cells, which stably express HSV-2 186 pUL21 [42], and HaCaT16 cells, which stably express HSV-2 186 pUL16 [30] were maintained in Dulbecco’s modified Eagle medium (DMEM) supplemented with 10% fetal bovine serum (FBS), penicillin/streptomycin and GlutaMax in a 5% CO_2_ environment. HSV-2 and HSV-1 strains deficient in pUL21 (**Δ**21) were constructed as described previously [31,42]. All pUL21 deficient viruses were propagated in HaCaT21 cells. Recombinant HSV-2 strain 186 containing mCherry (mCh) fused to the minor capsid protein VP26 and the corresponding Δ21 mutant have been described previously [31]. Times post-infection reported as hours post-infection (hpi) refers to the time elapsed following medium replacement after a one-hour inoculation period.

Two independently isolated HSV-1 KOS Δ21/Δ16 strains (HΔ21/Δ16 and EΔ21/Δ16) were constructed by CRISPR/Cas9 mutagenesis using UL16 guide RNAs reported previously [42]. Briefly, HSV-1 KOS Δ21 genomic DNA was co-transfected into 293T21 cells along with plasmids expressing UL16-specific gRNAs using the calcium phosphate co-precipitation method [65]. At 24 hours post-transfection, the medium was replaced with DMEM containing 2% FBS and 1% carboxymethylcellulose to enable plaque formation. Three days later, plaques were picked, and DNA was isolated from the picked plaques and screened for deletion of UL16 and UL21 by PCR. Viruses containing UL21 and UL16 deletions were analyzed by western blotting to confirm that pUL21 and pUL16 were not produced in infected cells. HSV-1 KOS HΔ21/Δ16 and EΔ21/Δ16 mutants were propagated on a 1:1 mixture of HaCaT21 and HaCaT16 cell monolayers.

### Immunological reagents and chemicals

A recombinant full-length HSV-2 186 GST-UL25 fusion protein was produced in *E*. *coli* strain BL21. Bacteria were lysed, and inclusion bodies were purified using a B-Per protein purification kit (Thermo Fisher Scientific, Ottawa, ON) according to the manufacturer’s instructions. Proteins in inclusion bodies were separated on preparative SDS-PAGE gels, the band corresponding to the glutathione *S*-transferase fusion excised and sent to Virusys (Taneytown, MD) to immunize a goat for pUL25 antiserum production. Goat polyclonal antiserum against HSV-2 pUL25 was used for western blotting at a dilution of 1:15000. Mouse anti-human IRF-3 (BD PharMingen, San Diego, CA) was used at a dilution of 1:100, and Alexa Fluor 568-conjugated donkey anti-mouse (Thermo Fisher Scientific, Ottawa, ON) was used at a dilution of 1:500 for indirect immunofluorescence microscopy. Mouse monoclonal antibody against HSV ICP5 (Virusys, Taneytown, MD) was used for western blotting and immunofluorescence at a dilution of 1:500. Mouse monoclonal antibody against β-actin (Sigma, St. Louis, MO) was used for western blotting at dilution of 1:2000. Rabbit polyclonal antisera against HSV pUL16 [34], a gift from Dr. J. W. Wills (Pennsylvania State University), was used for western blotting at a dilution of 1:3000. Mouse monoclonal antibody against HSV-1 pUL25 [13], a gift from Dr. F. L. Homa (University of Pittsburgh), was used for western blotting at a dilution of 1:2000. Chicken polyclonal antisera against HSV pUL17 [66], a gift from Dr. J. D. Baines (Cornell University), was used for western blotting at a dilution of 1:2500. Rat polyclonal antisera against HSV pUL21 [31] was used for western blotting at a dilution of 1:600. AF 488 picolyl azide and 5-ethynyl-2’-deoxycytidine (EdC) (Click Chemistry Tools, Scottsdale, AZ) were used for click-chemistry according to the manufacturer’s instructions.

### Transmission electron microscopy

Vero cells were plated on 100mm dishes one day prior to infection. Vero cells were infected at a multiplicity of infection (MOI) of 3 for 18 hours with WT strains, **Δ**21 mutants, or Δ21/Δ16 mutants. Infected cells were washed with PBS three times before fixing in 2.5% EM grade glutaraldehyde (Ted Pella, Redding, CA) in 0.1 M sodium cacodylate buffer (pH 7.4) for 60 minutes. Cells were collected by scraping into fixative and centrifugation at 300 x g for five minutes. Cell pellets were carefully enrobed in an equal volume of molten 5% low-melting temperature agarose (Lonza, Rockland, ME) and allowed to cool. Specimens in agarose were incubated in 2.5% glutaraldehyde in 0.1M sodium cacodylate buffer (pH 7.4) for 1.5 hours and post-fixed in 1% osmium tetroxide for one hour. The fixed cells in agarose were washed with distilled water three times and stained in 0.5% uranyl acetate overnight before dehydration in ascending grades of ethanol (30%-100%). Samples were transitioned from ethanol to infiltration with propylene oxide and embedded in Embed-812 hard resin (Electron Microscopy Sciences, Hatfield, PA). Blocks were sectioned at 50–60ηm and stained with uranyl acetate and Reynolds’ lead citrate. Images were collected using a Hitachi H-7000 transmission electron microscope. A-, B- and C-capsids were quantified in the nucleus (n = 9–12 micrographs per condition) and cytoplasm (n = 11–14 micrographs per condition), as well as in PEVs (n = 11–27 micrographs per condition). A total of 276–901 nuclear/cytoplasmic capsids (A-, B-, and C-capsids) per condition were quantified. A total of 143–176 extracellular virions per condition were quantified. A total of 7–158 PEVs were analyzed per condition.

### Evaluation of nuclear envelope integrity

Vero cells were infected with HSV-1 KOS WT or Δ21 strains at an MOI of 0.1. At 18 hpi cells were washed three times with PBS and fixed in 4% para-formaldehyde for 15 minutes at room temperature (RT). Samples were washed three times in PBS and permeabilized by adding 0.5% triton X-100 in PBS for 15 minutes at RT or by adding 0.01% saponin in PBS for 10 minutes at RT. Samples permeabilized with saponin had 0.01% saponin in all subsequent washes and antibody dilutants. Cells were stained using a mouse monoclonal HSV ICP5 antibody as described previously [30]. Images were captured with an Olympus FV1000 laser scanning confocal microscope using a 60X (1.42 NA) oil immersion objective lens and FV10 ASW 4.01 software.

### Preparation of EdC labelled viruses

To incorporate EdC into viral genomes, T12 cells were seeded onto 150mm dishes and grown to confluence. Three days after confluency was reached cells were infected at an MOI of 0.01. At 4 hpi, 5-ethynyl-2’-deoxycytidine (EdC) was added directly to the medium at a final concentration of 1μM and fresh EdC was added every 24 hours until complete cytopathic effect was observed. Cells were scraped into the medium and virus stocks were prepared as described previously [67]. Virus stocks were cleared of residual EdC using a PD-10 desalting column (GE Healthcare, Mississauga, ON) utilizing the manufacturer’s instructions. Desalted virus preparations were aliquoted and stored at -80°C.

### Nuclear translocation of IRF-3

WT viruses and their corresponding Δ21 mutants were used to infect T12 cells on ice at an MOI of 3. After one hour, the inoculum was replaced with complete medium containing 50μg/mL of cycloheximide. At 0, 2, 4 and 6 hpi, cells were washed three times with PBS and fixed by adding 4% para-formaldehyde in PBS for 15 minutes at room temperature. Cells were permeabilized and stained using a mouse monoclonal IRF-3 antibody as described previously [30]. As a positive control for IRF-3 nuclear translocation, T12 cells were transfected with 2μg poly I:C (InvivoGen, San Diego, CA) using X-treme GENE HP DNA transfection reagent (Roche, Laval, QC) following the manufacturer’s instructions. At 6 hours post-transfection, cells were fixed and stained for IRF-3.

### Capsid isolation

Capsid preparation was performed similarly to previously published methods [62]. Five 150mm dishes of confluent Vero cells were infected with each HSV WT, or their corresponding Δ21, strains. When complete CPE was reached, infected cells were harvested and centrifuged at 1,000 x g for 10 minutes at 4°C. Supernatants were discarded and cell pellets were resuspended in 50mL of cold PBS and pelleted at 1,000 x g for 10 minutes at 4°C. The supernatants were discarded, and cells were lysed by resuspension in 10mL cold NP-40 lysis buffer (150mM NaCl, 10mM Tris pH 7.2, 1% NP-40, 5mM DTT, 2mM MgCl_2_) containing protease inhibitors (Roche, Laval, QC) on ice for 30 minutes. The nuclei were then pelleted at 1,000 x g for 10 minutes at 4°C, supernatants were discarded, and nuclei were resuspended in 3mL of TNE buffer (150mM NaCl, 20mM Tris pH 7.5, 1mM EDTA). Nuclei were then broken by successive passages through 18-, 22- and 25-gauge syringe needles. Nuclear lysates were treated with 1μL of benzonase (250U/μL), incubated for 15 minutes at RT and then clarified by centrifugation at 3,000 x g for 10 minutes at 4°C. Clarified nuclear lysates were layered onto a 2mL 35% (w/v) sucrose cushion prepared in TNE and centrifuged at 35,000 rpm for 32 minutes at 4°C in a Beckman SW55 rotor. Pellets containing nucleocapsids were resuspended in 250μL of TNE buffer and sonicated briefly using a chilled cup-horn sonicator. Capsids were then layered onto 10mL 20% to 50% linear sucrose gradients prepared using a Gradient Master (BioComp, Fredericton, NB) and centrifuged in a Beckman SW41 rotor at 25,000 rpm for 1 hour. After centrifugation, distinct light-scattering bands representing A-, B-, and C-capsids were evident. For capsids to be analyzed by western blotting, gradients were fractioned from the top of the tube into 1mL fractions. For capsids analyzed by mass spectrometry, capsids were isolated from the gradient by side wall puncture using a 16 ½-gauge needle. Samples collected from the gradient were diluted with 0.5 volumes of TNE buffer and capsids pelleted at 26,000 rpm for 30 minutes at 4°C in a Beckman MLA130 rotor. Supernatants were discarded and capsid pellets were resuspended in 35μL of 1X SDS-PAGE loading buffer and stored at -20°C until analyzed by western blotting or mass spectrometry.

### Mass spectrometry of nuclear capsids

HSV-1 KOS WT and Δ21 nuclear A-, B-, and C-capsids were electrophoresed 0.5 cm into a 9% SDS-PAGE gel. Protein bands from each sample were visualized by staining with SimplyBlue (Thermo Fisher Scientific, Ottawa, ON). Proteins were excised in a 0.5cm by 0.5cm slab of acrylamide. Slabs were placed in 2mL microcentrifuge tubes containing 500μL of 1% acetic acid and sent on wet ice to the Southern Alberta Mass Spectrometry Facility for liquid chromatography tandem mass spectrometry. Proteins identified by mass spectrometry that met the following criteria were included in the analysis: a >95% protein and peptide threshold, a minimum number of ≥3 unique peptides identified, and by removing common contaminants (e.g., keratins). To ensure direct comparisons could be made between WT and Δ21 samples, Δ21 A-, B-, and C-capsids were normalized to respective WT capsids using total spectrum counts for VP5. Three biological replicates were analyzed for each strain.

### Western blot analysis

Proteins were electrophoresed through 8% or 10% SDS-PAGE gels. Proteins were transferred onto polyvinylidene fluoride (PVDF) membranes and blocked for 1 hour in Intercept tris-buffered saline (TBS) blocking buffer (LI-COR, Lincoln, NE). Membranes were probed with appropriate dilutions of antibodies (VP5, pUL25, pUL17, pUL21, or pUL16) overnight at 4°C. Membranes were washed three times with Tris-buffered saline (10mM Tris pH 8.0, 150mM NaCl) containing 0.1% Tween20 (TBST) for seven minutes and then appropriate dilutions of secondary antibodies were added to the membranes and incubated at RT for one hour. Membranes were then washed three times with TBST for seven minutes and finally with TBS before imaging on an Odyssey CLx imaging system (LI-COR, Lincoln, NE). Images and signal intensities of viral proteins were obtained using Image Studio Lite software version 5.2.5. VP5 signals were used to normalize WT and Δ21 capsid numbers. Normalized signal intensity for pUL21, pUL16, pUL25 and pUL17 in Δ21 A-, B-, and C-capsid samples were directly compared to corresponding WT capsids samples.

## Supporting information

S1 Table Results of mass spectrometry analysis of A-, B- and C-capsids isolated from nuclei of HSV-1 (KOS) WT and Δ21 infected cells. Molecules of particular interest are highlighted in yellow.
(XLSX)
